# Another Year for *CFG*

**DOI:** 10.1002/cfg.341

**Published:** 2003-12

**Authors:** Steve Oliver


					Comparative and Functional Genomics
Comp Funct Genom 2003; 4: 570.
Published online in Wiley InterScience (www.interscience.wiley.com). DOI: 10.1002/cfg.341
Editorial
Another year for CFG
Overall, it has been a good year for CFG, with
some excellent research papers, and interesting
collections of reviews in our special `Ontologies'
issue, the extended coverage of the Plant and Ani-
mal Genomes Conference, and the special sections
from the ESF Structural Genomics workshop and
the ESF Functional Genomics and Disease 2003
Conference. In fact, such is the demand for page
space in CFG that we will be increasing to 8 issues
a year in 2004.
There was some sad news however, when we
heard that Andrei Mirzabekov had passed away this
June. He was a member of our editorial board from
the launch of the journal. This is a great loss for
genomics and for Russian molecular biology, and
we would like to extend our sympathy to his family
and friends.
Steve Oliver
Editor-in-Chief
Copyright  2003 John Wiley & Sons, Ltd.

				

